# Facedown Positioning Following Surgery for Large Full-Thickness Macular Hole: A Multicenter Randomized Clinical Trial

**DOI:** 10.1001/jamaophthalmol.2020.0987

**Published:** 2020-05-07

**Authors:** Saruban Pasu, Lauren Bell, Zohra Zenasni, Doris Lanz, Irene A Simmonds, Ann Thompson, David Yorston, D. Alistair H. Laidlaw, Catey Bunce, Richard Hooper, James W. B. Bainbridge

**Affiliations:** 1 National Institute of Health Research Biomedical Research Centre (BRC), Moorfields Eye Hospital National Health Service (NHS) Foundation Trust, UCL Institute of Ophthalmology, London, England; 2 Moorfields Eye Hospital NHS Foundation Trust, London, England; 3 UCL Institute of Ophthalmology, London, England; 4 Pragmatic Clinical Trial Unit, Queen Mary University of London, London, England; 5 Blizard Institute, Barts and The London School of Medicine and Dentistry, London, England; 6 Gartnavel Royal Hospital, Glasgow, Scotland; 7 St Thomas' Hospital, London, England; 8 Department of Primary Care and Public Health Sciences, King’s College London, London, England

## Abstract

**Importance:**

The value of facedown positioning following surgery for large full-thickness macular holes is unknown.

**Objective:**

To determine whether advice to position facedown postoperatively improves the outcome for large macular holes.

**Design, Setting, and Participants:**

This randomized, parallel group superiority trial with 1:1 randomization stratified by site with 3 months’ follow-up was conducted at 9 sites across the United Kingdom and included participants with an idiopathic full-thickness macular hole of at least 400 μm minimum linear diameter and a duration of fewer than 12 months. All participants had vitrectomy surgery with peeling of the internal limiting membrane and injection of perfluoropropane (14%) gas, with or without simultaneous surgery for cataract.

**Interventions:**

Following surgery, participants were randomly advised to position either facedown or face forward for 8 hours daily for 5 days.

**Main Outcomes and Measures:**

The primary outcome was closure of the macular hole determined 3 months following surgery by masked optical coherence tomography evaluation. Secondary outcome measures at 3 months were visual acuity, participant-reported experience of positioning, and quality of life measured by the National Eye Institute Visual Function Questionnaire 25.

**Results:**

A total of 185 participants (45 men [24.3%]; 156 white [84.3%]; 9 black [4.9%]; 10 Asian [5.4%]; median age, 69 years [interquartile range, 64-73 years]) were randomized. Macular hole closure was observed in 90 (85.6%) who were advised to position face forward and 88 (95.5%) advised to position facedown (adjusted odds ratio, 3.15; 95% CI, 0.87-11.41; *P* = .08). The mean (SD) improvement in best-corrected visual acuity at 3 months was 0.34 (0.69) logMAR (equivalent to 1 Snellen line) in the face-forward group and 0.57 (0.42) logMAR (equivalent to 3 Snellen lines) in the facedown group (adjusted mean difference, 0.22 [95 % CI, 0.05-0.38]; equivalent to 2 Snellen lines); 95% CI, 0.05-0.38; *P* = .01). The median National Eye Institute Visual Function Questionnaire 25 score was 89 (interquartile range, 76-94) in the facedown group and 87 (interquartile range, 73-93) in the face-forward group (mean [SD] change on a logistic scale, 0.08 [0.26] face forward and 0.11 [0.25] facedown; adjusted mean [SD] difference on a logistic scale, 0.02; 95% CI, −0.03 to 0.07; *P* = .41).

**Conclusions and Relevance:**

The results do not prove that facedown positioning following surgery is more likely to close large macular holes compared with facing forward but do support the possibility that visual acuity outcomes may be superior.

**Trial Registration:**

Isrctn.org Identifier: 12410596

## Key Points

**Question:**

Is the closure of large macular holes improved by advising facedown positioning postsurgery?

**Findings:**

In this randomized clinical trial of 185 participants, macular hole closure in those advised to position facing down was not superior to macular hole closure in those facing forward.

**Meaning:**

The results do not prove that facedown positioning following surgery is more likely to close large macular holes.

## Teaser

This randomized clinical trial examines the effect of postoperative facedown positioning on outcomes for large macular holes.

## Introduction

Idiopathic full-thickness macular holes cause severe sight impairment, with an incidence of 8 per 100 000 individuals per year.^1^ The condition accounts for 0.2 to 3.3 per 1000 of those affected by sight impairment^2,3^ and is more common in older people.^2^ It occurs as a consequence of age-related degeneration of the vitreous gel, resulting in traction at the central macula and focal dehiscence of the neurosensory retina. The retina at the edges of a full-thickness macular hole typically becomes separated from the underlying pigment epithelium by subretinal fluid and swollen by the accumulation of intraretinal fluid.

Full-thickness macular holes are conventionally managed by surgical removal of the vitreous gel to relieve any persistent traction acting at the macula and intraocular injection of a gas bubble to provide a temporary scaffold that promotes hole closure. Following the surgical procedure, a period of facedown positioning may be advised to improve the outcome by maintaining consistent close contact of the gas bubble with the macula at the posterior pole. However, facedown positioning can be arduous, uncomfortable, and disabling^4^; it is of unproven benefit and presents a risk of harm.^5^ A systematic review in 2011 found that, for macular holes smaller than 400 μm in minimum linear diameter, the estimated association of facedown positioning with hole closure was not statistically significant.^6,7^ For macular holes larger than 400 μm minimum linear diameter, the evidence from randomized clinical trials suggested that postoperative positioning may improve the rate of hole closure.^6,8,9^ However, the evidence was insufficient to draw firm conclusions with which to guide practice because it was based on fewer heterogeneous studies, with the use of several different tamponade gases within a single study, and lacking patient-reported outcomes. A subsequent large retrospective nonrandomized noninferiority study did not exclude the possibility of benefit.^10^ The aim of this study was to determine whether advice to position facedown postoperatively improves the outcome of surgery for large (≥400 μm) full-thickness macular holes.

## Methods

### Consent and Ethics

The research adhered to the tenets of the Declaration of Helsinki. The trial was approved by the national ethics committee and registered with ISRCTN (12410596) and the UK Clinical Research Network Portfolio (17966). Participants gave their fully informed written consent before enrollment.

### Design

The detailed methods are described in the published protocol (Supplement 1).^11^ We performed a multicenter interventional parallel group superiority comparative randomized clinical trial comparing facedown positioning with face-forward positioning, with 1:1 randomization stratified by site. Consolidated Standards of Reporting Trials guidelines were followed.

### Participants

We included participants with an idiopathic full-thickness macular hole of at least 400-μm minimum linear diameter and a duration of fewer than 12 months who elected to have surgery for macular hole with or without simultaneous surgery for cataract. All participants had vitrectomy surgery with peeling of the inner limiting membrane and injection of perfluoropropane (C3F8), 14%, gas, with or without simultaneous surgery for cataract. No additional intervention (such as inner limiting membrane flap) was performed. If postoperative positioning was advised to support a retinal tear identified during surgery, participants were excluded before randomization.

### Intervention

Following surgery, participants were randomly advised to position either facedown or face forward for at least 8 consecutive or nonconsecutive hours daily for 5 days. All participants were advised to avoid a faceup position for 5 days. Positioning was explained with the help of written instructions and diagrams.

### Outcomes

The primary outcome was anatomical closure of the macular hole at 3 months following surgery determined by spectral-domain optical coherence tomography (OCT) evaluation. Two independent retinal specialists, masked to treatment allocation, graded independently the outcome in each instance as closed, open and flat (without a cuff of subretinal fluid), or open and elevated (with a cuff of subretinal fluid). The categories open and flat and open and elevated were combined into a single category of open for analysis.

The secondary outcome measures at 3 months were best-corrected visual acuity (BCVA) measured using a Snellen chart at a standard distance of 6 m, participant-reported experience of positioning on a scale from 0 (very difficult) to 10 (very easy), and participant-reported health and quality of life evaluated using the National Eye Institute Visual Function Questionnaire 25 (NEI VFQ-25) from 0 (worst health and quality of life) to 100 (best health and quality of life). We also investigated the participants’ own judgments of their individual outcomes by asking each the question “Given what you now know, would you still have elected to have the operation?”

### Randomization and Masking

Participants were randomly advised, in a 1:1 ratio, to position either face forward or facedown. The randomization was stratified by site using random permuted blocks of size 4 or 6 in equal proportions. The randomization was performed using a secure bespoke online randomization service implemented by the Pragmatic Clinical Trials Unit. Randomization was performed following surgery to ensure the masking of the surgeon to the treatment allocated. The independent retina specialists responsible for grading OCT scans were also masked to the treatment allocation. The participants themselves and the clinical teams managing their care were unmasked.

### Statistical Considerations and Sample Size

The sample size calculation and statistical analysis plan are described in Supplement 2.^12^ Clinical consensus was that facedown positioning would be recommended if this was to improve the rate of success by 15%. This was the smallest clinically relevant treatment difference that we sought to detect. Previous findings indicated that surgery for large macular holes (≥400-μm diameter) without advice to position facedown results in anatomical hole closure in 80%.^6^ To detect a 15% difference in outcomes with 85% power and 95% confidence, we sought to include 86 participants in each of the 2 treatment groups. With an anticipated 10% loss to follow-up, we aimed to recruit 96 participants to each group. Dichotomous outcomes were analyzed by mixed logistic regression and continuous outcomes by mixed linear regression. Analyses were adjusted for the fixed effects of macular hole size and phakic lens status at baseline and a random effect of site. The fitted logistic regression model for the primary outcome, which estimates the treatment effect as an odds ratio, was also used to calculate absolute risk differences for particular covariate values. A logistic transformation (log(*x*/[1-*x*]) was applied to NEI VFQ-25 scores to give them a less skewed distribution. Further details of analyses are given in the published analysis plan.^12^ *P* values were 2-tailed with no correction for multiple analyses with statistical significance at .05. Analyses were conducted with Stata, version 14.2 (StataCorp).

## Results

### Baseline Characteristics

A total of 206 participants were enrolled in the study (Figure). Of these, 22 withdrew before randomization because they no longer met the inclusion criteria or no longer wished to participate. A total of 185 participants were randomized (Table 1); of these, 1 participant (0.5%) withdrew before treatment allocation and 3 from each group (3.2%) were excluded following randomization because they were found to be ineligible owing to a macular hole dimension of less than 400-μm minimum linear diameter. No participant was lost to follow-up. The group advised to position facedown included more black participants and fewer Asian participants and had a slightly smaller median macular hole diameter. The baseline characteristics of the 2 groups otherwise appeared similar.

**Figure.**
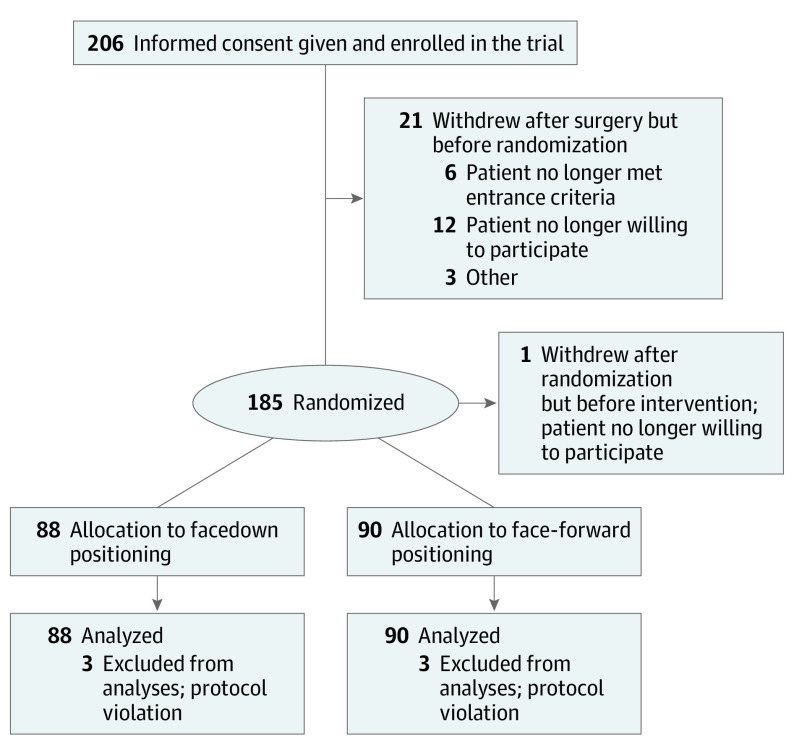
CONSORT Diagram

**Table 1.** Baseline Characteristics

Characteristic	Positioning, No. (%)	
	Face-forward (n = 90)	Facedown (n = 88)
Age, median (IQR), y	69 (64-73)	69 (64-73)
Male sex	22 (24.4)	23 (26.1)
Race/ethnicity		
White	78 (86.7)	78 (88.6)
Black	2 (2.2)	7 (8.0)
Asian	8 (8.9)	2 (2.3)
Mixed	0 (0.0)	1 (1.1)
Other	2 (2.2)	0 (0.0)
Laterality, left side	47 (52.2)	49 (55.7)
Duration of symptoms, median (IQR), mo	5 (3-7)	5 (4-7)
BCVA, median (IQR)	20/200 (20/80-20/200)	20/200 (20/80-20/200)
Lens status, phakic	78 (86.7)	72 (81.8)
Cataract surgery performed	44 (48.9)	45 (51.1)
Macular hole diameter on OCT, median (IQR)	517 (460-588)	480 (446-557)
Quality of life VFQ-25, mean (SD)	77.1 (17.4)	76.4 (17.9)
Vitreofoveal detachment present	32 (35.6)	33 (37.5)

Abbreviations: BCVA, best-corrected visual acuity; IQR, interquartile range; OCT, optical coherence tomography; VFQ-25, Visual Function Questionnaire 25.

### Outcomes

#### Primary Outcome

The grading of macular hole status on OCT scans at 3 months was consistent between the 2 masked graders in every instance. In 1 instance, in the absence of an OCT scan, the outcome (open hole) was determined by unmasked clinical examination. Successful macular hole closure was observed in 77 (85.6%) of those advised to position face forward and in 84 participants (95.5%) advised to position facedown (adjusted odds ratio, 3.15; 95% CI, 0.87-11.41; *P* = .08). Hole size (but not lens status) was strongly associated with risk, and consequently when the odds ratio is translated to an absolute risk difference scale, this risk difference is dependent on macular hole size (but not on lens status) (Table 2). At the median macular hole size (488.5 μm; interquartile range [IQR], 450-578), the odds ratio of 3.15 corresponded to an absolute risk difference of 4.1% (95% CI, −0.8% to 9.1%), or a number needed to treat of 24.

**Table 2.** Effect of Macular Hole Size on Closure

Macular hole size	μm	Successful closure of macular hole, absolute risk difference (95% CI)	*P* value	NNT
Minimum value	400	0.018 (−0.008 to 0.044)	.18	55
Lower quartile	450	0.029 (−0.009 to 0.067)	.13	34
Median	488.5	0.041 (−0.008 to 0.091)	.10	24
Upper quartile	578	0.089 (−0.004 to 0.182)	.06	11
Maximum value	854	0.273 (−0.039 to 0.586)	.09	4

Abbreviation: NNT, number needed to treat.

#### Secondary Outcomes

The mean (SD) logMAR-converted BCVA at 3 months was 0.87 (0.57) (Snellen equivalent, 20/160 OU) in the face-forward group and 0.68 (0.39) (Snellen equivalent, 20/100 OU) in the facedown group (adjusted mean difference, 0.16; 95% CI, 0.02-0.30; *P* = .02) (Table 3). The mean (SD) improvement in BCVA at 3 months was 0.34 (0.69) logMAR (equivalent to 1 Snellen line) in the face-forward group and 0.57 (0.42) logMAR (equivalent to 3 Snellen lines) in the facedown group. The adjusted mean difference in mean improvement in logMAR acuity was 0.22 (equivalent to 2 Snellen lines) (95% CI, 0.05-0.38; *P* = .01). In a post hoc analysis of visual acuities, we found that deterioration by 0.3 logMAR (15 Early Treatment Diabetic Retinopathy Study letters) or more affected 90 participants (12%) positioning face forward but only 88 (1%) of those positioning facedown (*P* = .01) (eTable in Supplement 3).

**Table 3.** Visual Acuity

Visual acuity	Positioning, mean (SD)		Regression coefficient (95% CI)	*P* value
	Face-forward (n = 90)	Facedown (n = 88)		
Best-corrected visual acuity at 3 mo: logMAR conversion of Snellen acuity at 6 m^a^	0.87 (0.57)	0.68 (0.39)	0.16 (0.03 to 0.30)	.02
Change in logMAR conversion of best-corrected visual acuity using standard Snellen chart at 6 mo from baseline to 3 mo	−0.34 (0.69)	−0.57 (0.42)	0.22 (0.05 to 0.38)	.01

^a^ Model was fitted using xtreg adjusted for macular hole size and phakic lens status as well as the secondary outcome at baseline with site as a random effect as per the analysis plan.

The median participant-reported ease-of-positioning score (using a 10-point scale in which 0 was very difficult and 10 was very easy) was 9 (IQR, 7-10) in the face-forward group and 6 (IQR, 4-8) in the facedown group (*P* < .01) (Table 3). The proportion of participants reporting a score of 5 or more was 92.7% in the face-forward group and 56.1% in the facedown group (adjusted odds ratio, 0.10; 95% CI, 0.04-0.27; *P* < .001) (Table 4). The proportion of participants reporting at 3 months that, given their experience, they would still have elected to have the operation was 90.5% in the face-forward group and 90.4% in the facedown group (adjusted odds ratio, 1.01; 95% CI, 0.36-2.88; *P* = .98). The median NEI VFQ-25 score was 87 (IQR, 73-93) in the face-forward group and 89 (IQR, 76-94) in the facedown group (adjusted mean difference on a logistic scale, 0.02; 95% CI, −0.03 to 0.07; *P* = .41). There were no related unexpected serious adverse events.

**Table 4.** Participant-Reported Outcomes^a^

Outcomes	Positioning, No. (%)		Odds ratio (95% CI)	*P* value
	Face-forward (n = 90)	Facedown (n = 88)		
No. of participants with persistently open macular hole electing to proceed with further surgery^b^	10 (76.9)	4 (100.0)	NA	
Dichotomized participant-reported experience of positioning at 3 mo^c^	76 (92.7)	46 (56.1)	9.75 (3.76-25.30)	<.01
Participant-reported outcome if they would still have elected to have the operation	NA			
Yes	76 (90.5)	75 (90.4)	0.99 (0.35-2.81)	.98
Yes/do not know (sensitivity analysis)	79 (94.0)	78 (94.0)	1.01 (0.28-3.71)	.98

Abbreviation: NA, not available.

^a^ Models were fitted using xtlogit adjusted for macular hole size and phakic lens status with site as a random effect as per the analysis plan.

^b^ Face-forward positioning (n = 13); facedown positioning (n = 4).

^c^ Patient-reported outcome was dichotomized as negative for answers (0-5) and positive for answers (6-10) after analysis of masked results through a histogram as per the analysis plan.

## Discussion

Surgical approaches for macular hole repair share key common techniques but also include variations that can confound the interpretation of outcomes unless appropriately controlled. Our trial was designed to determine the effect of positioning as it is commonly advised by many UK retina surgeons considering their preferred practice as determined by a survey of members of the British and Eire Association of Vitreoretinal Surgeons and their judgment of clinical equipoise. In this way, we could ensure efficient recruitment to the trial and generate results that were directly relevant to common practice. Our findings are applicable specifically to surgery for macular holes at least 400-μm minimum linear diameter with the use of perfluoropropane, 14%, gas and positioning facedown 8 hours daily for 5 days; the findings are not directly relevant to the use of alternative tamponade agents or positioning regimens. We elected to compare facedown positioning not with free positioning but with seated face-forward positioning so as to mitigate a perceived risk of harm from physical overactivity; the relative immobility of the seated position may reduce shear stress associated with intraocular fluid currents otherwise induced by physical activity in gas-filled eyes.^13^ We chose hole closure as the primary outcome on the advice of our lay advisory group, which shared a particular concern about the prospect of further intervention that might be necessary to close a macular hole that was persistently open despite surgery. In this randomized clinical trial of 185 participants, macular hole closure in those advised to position facing down was not shown to be superior to macular hole closure in those facing forward. However, secondary visual acuity outcomes appeared to be superior in the facedown group.

### Limitations

The study has several limitations. Participants were not provided with specific advice regarding positioning while sleeping because our lay advisory group judged that compliance while sleeping would be unfeasible for many people; a possible confounding effect cannot be excluded despite the randomized trial design. We chose not to estimate the adherence of participants with the advice to position postoperatively because such measurement is of unknown reliability and could influence behavior artificially. Instead we sought to determine pragmatically the effect of the advice to position as described. Given that trial participants reported difficulty with facedown positioning, the compliance of those advised to position facedown may have been poorer than those advised to position face forward. However, in clinical practice, the effect on compliance is likely to be in a similar direction. Because the trial was powered statistically to detect a difference in success rate of 15%, an effect size of less than 15% is not excluded. We chose to describe the effect in terms of the odds ratio, which may overestimate the risk compared with the risk ratio. The apparent change in risk difference with hole size is a consequence of the mathematical conversion from a log odds scale to a risk difference scale (an expression of our findings about the treatment effect from the logistic regression) and is not considered evidence of an interaction. We are not able to determine definitively whether the benefit to visual acuity is a consequence of hole closure owing to the limited size of the trial and its design. Because the visual acuity of the participants' contralateral nonoperated eyes was not collected, we are not able to interpret the differences between groups for the NEI VFQ-25 data regarding the better seeing eye, which can substantially influence this measure in retinal disease.^14^ The study was designed to determine the effect of positioning in primary surgery for macular holes and does not enable the evaluation of outcomes following further surgery for the few macular holes persistently open despite primary surgery.

## Conclusions

On the evidence of the findings, people with macular holes of a diameter of 400 μm or greater can be informed that surgery using the technique described and positioning face forward offers an estimated 86% likelihood of hole closure. The findings do not provide definitive evidence that the advice to position facedown improves the outcome for macular hole closure or visual acuity. The findings of prespecified and post hoc analyses suggest a modest benefit to visual acuity at 3 months, which was one of several secondary outcomes. In the absence of definitive evidence of an effect on hole closure, a possible benefit to visual acuity is unexplained, although facedown positioning might protect phakic eyes against gas-induced cataract. For people with macular holes of a diameter of 400 μm or greater, the results of this trial provide evidence to predict the likely outcome of surgery and guide their choice of positioning postoperatively.

## Notes

Supplement 1. Statistical Analysis Plan

Supplement 2. Trial Protocol

Supplement 3. eTable. Best-corrected visual acuity outcomes: post-hoc analyses

Supplement 4. Data sharing statement

## Notes

Journal Club Slides
