# Supplementary material for: Facedown Positioning Following Surgery for Large Full-Thickness Macular Hole: A Multicenter Randomized Clinical Trial
Source: JAMA Ophthalmol. 2020 May 7;138(7):725–30. doi: 10.1001/jamaophthalmol.2020.0987 (PMC7206530; doi:10.1001/jamaophthalmol.2020.0987)
Supplement: Supplement 3. — eTable. Best-corrected visual acuity outcomes: post-hoc analyses [file jamaophthalmol-138-725-s003.pdf]

Supplementary Online Content

Pasu S, Bell L, Zenasni Z, et al; Positioning in Macular Hole Surgery Study Group. Facedown positioning following surgery for large full-thickness macular hole: a multicenter randomized clinical trial. *JAMA Ophthalmol*. Published online May 7, 2020. doi:10.1001/jamaophthalmol.2020.0987

eTable. Best-corrected visual acuity outcomes: post-hoc analyses

This supplementary material has been provided by the authors to give readers additional information about their work.

e Table 1: Best-corrected visual acuity outcomes: post-hoc analyses

	Face-forward positioning	Face-down positioning	Odds Ratio (95% CI)	P-value
Proportion of participants whose BCVA improved by 0.3 LogMAR (15 ETDRS letters) or more - n (%) [N]	52 (57.8) [90]	64 (72.7) [88]	0.52 (0.26,1.04)	.06
Proportion of participants whose BCVA deteriorated by 0.3 LogMAR (15 ETDRS letters) or more - n (%) [N]	11 (12.2) [90]	1 (1.1) [88]	14.42 (1.75,118.68)	.01
Proportion of participants whose BCVA at 3 months was 20/40 Snellen or better (LogMAR less than or equal to 0.3) - n (%) [N]	7 (7.8) [90]	13 (14.8) [88]	0.53 (0.20,1.44)	.21
Proportion of participants whose BCVA at 3 months was 20/200 Snellen or poorer (LogMAR greater than or equal to 1) - n (%) [N]	34 (37.8) [90]	26 (30.0) [88]	1.29 (0.65,2.54)	.47

Note: Model fitted using xtlogit adjusted for macular hole size and phakic lens status with site as a random effect.

© Pasu et al. *JAMA Ophthalmology*.
