# Supplementary material for: Facedown Positioning Following Surgery for Large Full-Thickness Macular Hole: A Multicenter Randomized Clinical Trial
Source: JAMA Ophthalmol. 2020 May 7;138(7):725–30. doi: 10.1001/jamaophthalmol.2020.0987 (PMC7206530; doi:10.1001/jamaophthalmol.2020.0987)
Supplement: Supplement 4. — Data sharing statement [file jamaophthalmol-138-725-s004.pdf]

Data Sharing Statement

Pasu. Face-Down Positioning Following Surgery for Large Full-Thickness Macular Hole. *JAMA Ophthalmol*. Published May 07, 2020. 10.1001/jamaophthalmol.2020.0987

Data
Data available: Yes
Data types: Deidentified participant data
How to access data: j.bainbridge@ucl.ac.uk
When available: With publication

Supporting Documents
Document types: Statistical/analytic code
How to access documents: PROTOCOL https://www.springermedizin.de/pims-positioning-in-macular-hole-surgery-trial-a-multicentre-int/9782730 SAP https://www.springermedizin.de/positioning-in-macular-hole-surgery-pims-statistical-analysis-pl/12441960
When available: With publication

Additional Information
Who can access the data: to researchers whose proposed use of the data has been approved
Types of analyses: for a specified purpose
Mechanisms of data availability: after approval of a proposal
