# Supplementary material for: Facedown Positioning Following Surgery for Large Full-Thickness Macular Hole: A Multicenter Randomized Clinical Trial
Source: JAMA Ophthalmol. 2020 May 7;138(7):725–30. doi: 10.1001/jamaophthalmol.2020.0987 (PMC7206530; doi:10.1001/jamaophthalmol.2020.0987)
Supplement: Journal Club Slides [file jamaophthalmol-138-725-slides.pptx]

## Slide 1
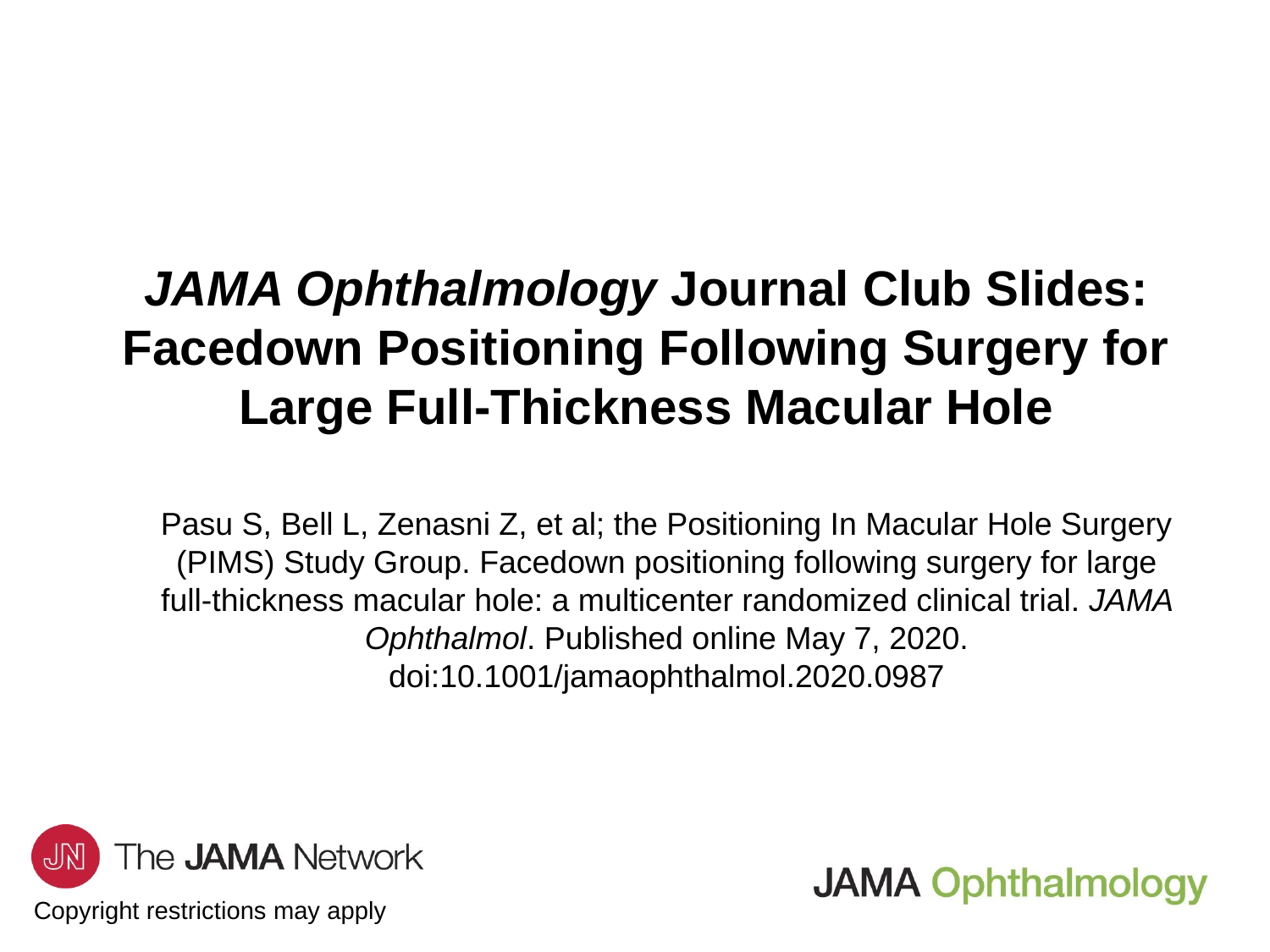

JAMA Ophthalmology Journal Club Slides:
Facedown Positioning Following Surgery for Large Full-Thickness Macular Hole
Pasu S, Bell L, Zenasni Z, et al; the Positioning In Macular Hole Surgery (PIMS) Study Group. Facedown positioning following surgery for large full-thickness macular hole: a multicenter randomized clinical trial. JAMA Ophthalmol. Published online May 7, 2020. doi:10.1001/jamaophthalmol.2020.0987

## Slide 2
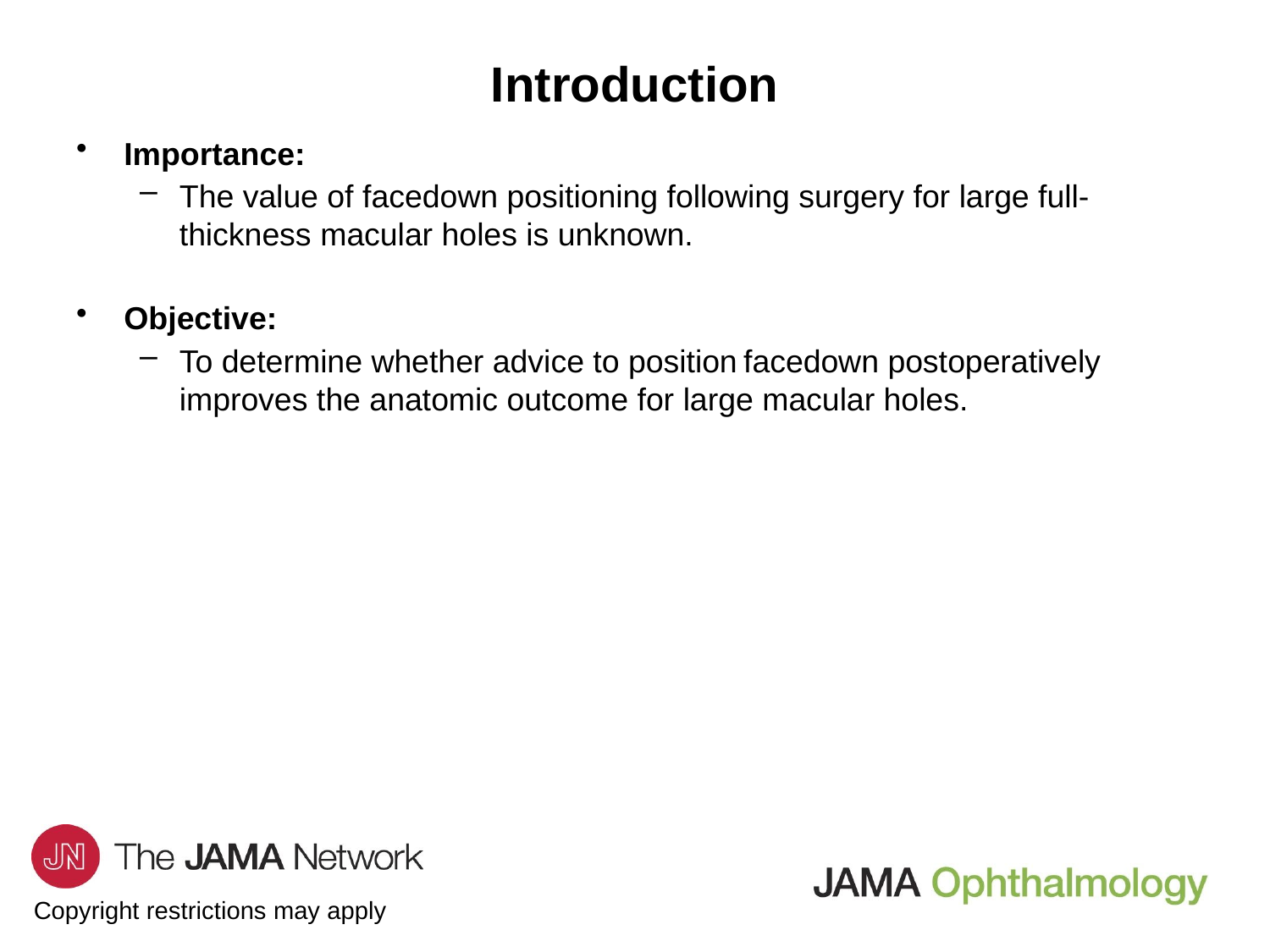

# Introduction
Importance:
The value of facedown positioning following surgery for large full-thickness macular holes is unknown.
Objective:
To determine whether advice to position facedown postoperatively improves the anatomic outcome for large macular holes.

## Slide 3
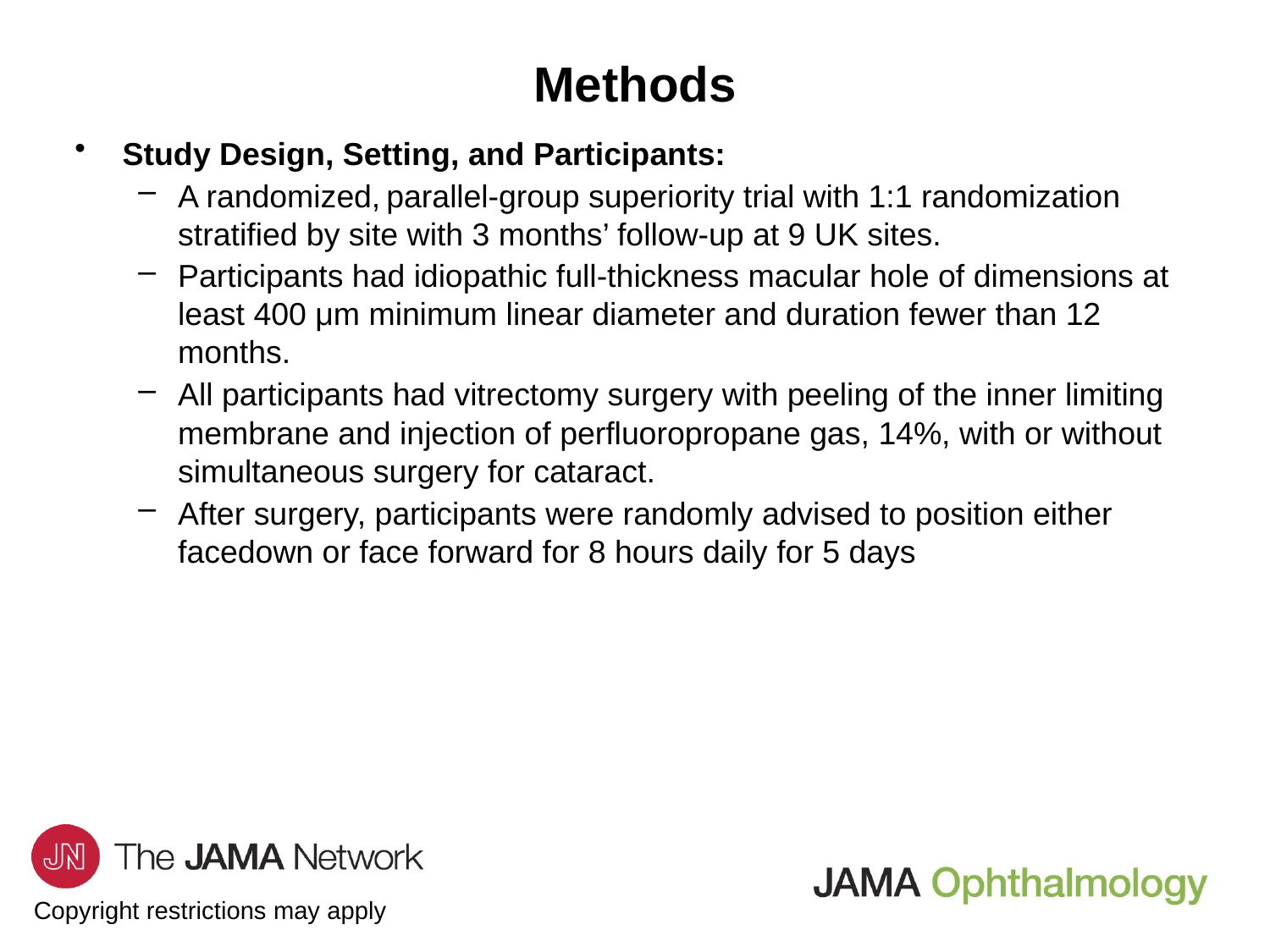

# Methods
Study Design, Setting, and Participants:
A randomized, parallel-group superiority trial with 1:1 randomization stratified by site with 3 months’ follow-up at 9 UK sites.
Participants had idiopathic full-thickness macular hole of dimensions at least 400 μm minimum linear diameter and duration fewer than 12 months.
All participants had vitrectomy surgery with peeling of the inner limiting membrane and injection of perfluoropropane gas, 14%, with or without simultaneous surgery for cataract.
After surgery, participants were randomly advised to position either facedown or face forward for 8 hours daily for 5 days

## Slide 4
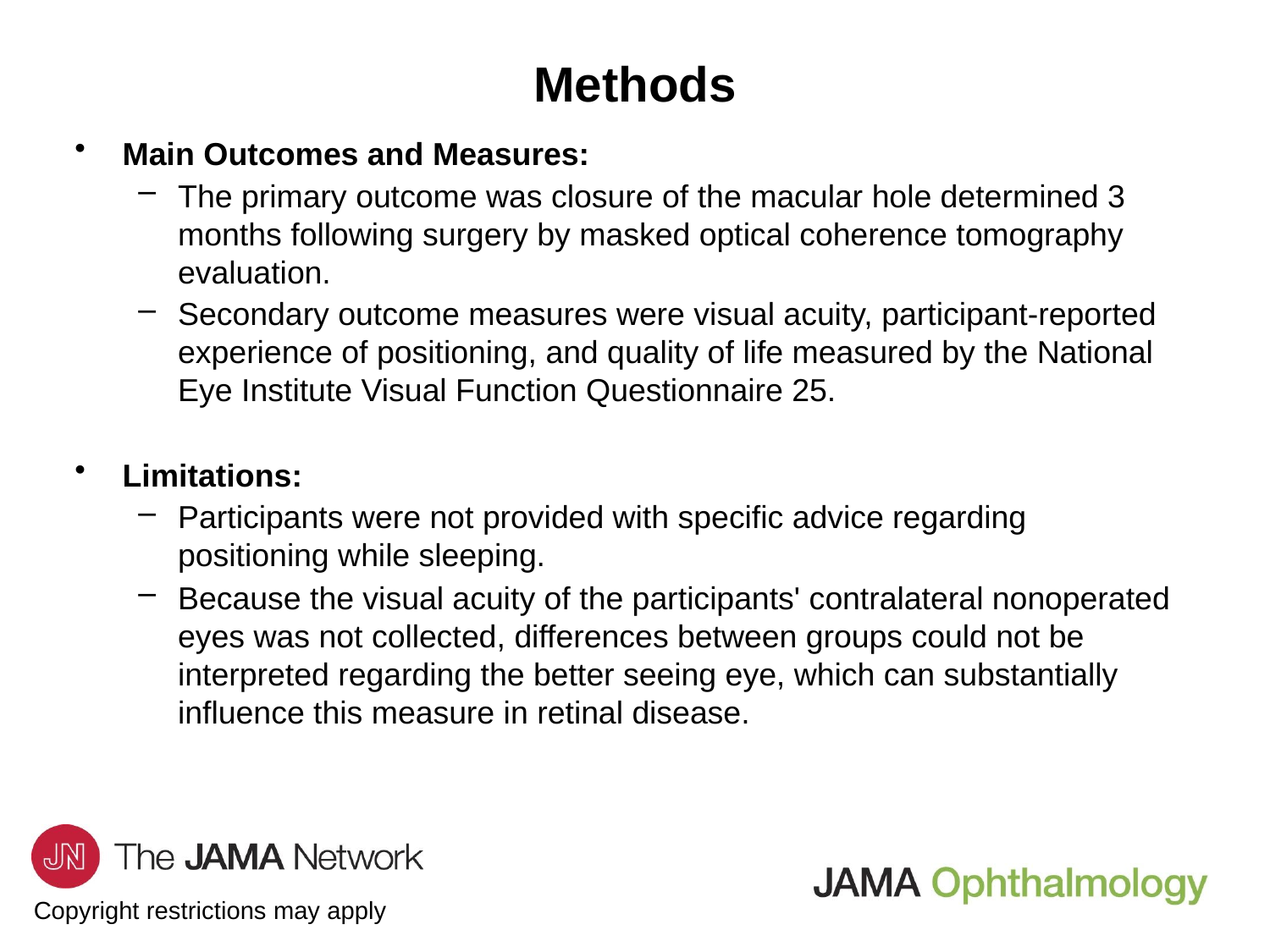

# Methods
Main Outcomes and Measures:
The primary outcome was closure of the macular hole determined 3 months following surgery by masked optical coherence tomography evaluation.
Secondary outcome measures were visual acuity, participant-reported experience of positioning, and quality of life measured by the National Eye Institute Visual Function Questionnaire 25.
Limitations:
Participants were not provided with specific advice regarding positioning while sleeping.
Because the visual acuity of the participants' contralateral nonoperated eyes was not collected, differences between groups could not be interpreted regarding the better seeing eye, which can substantially influence this measure in retinal disease.

## Slide 5
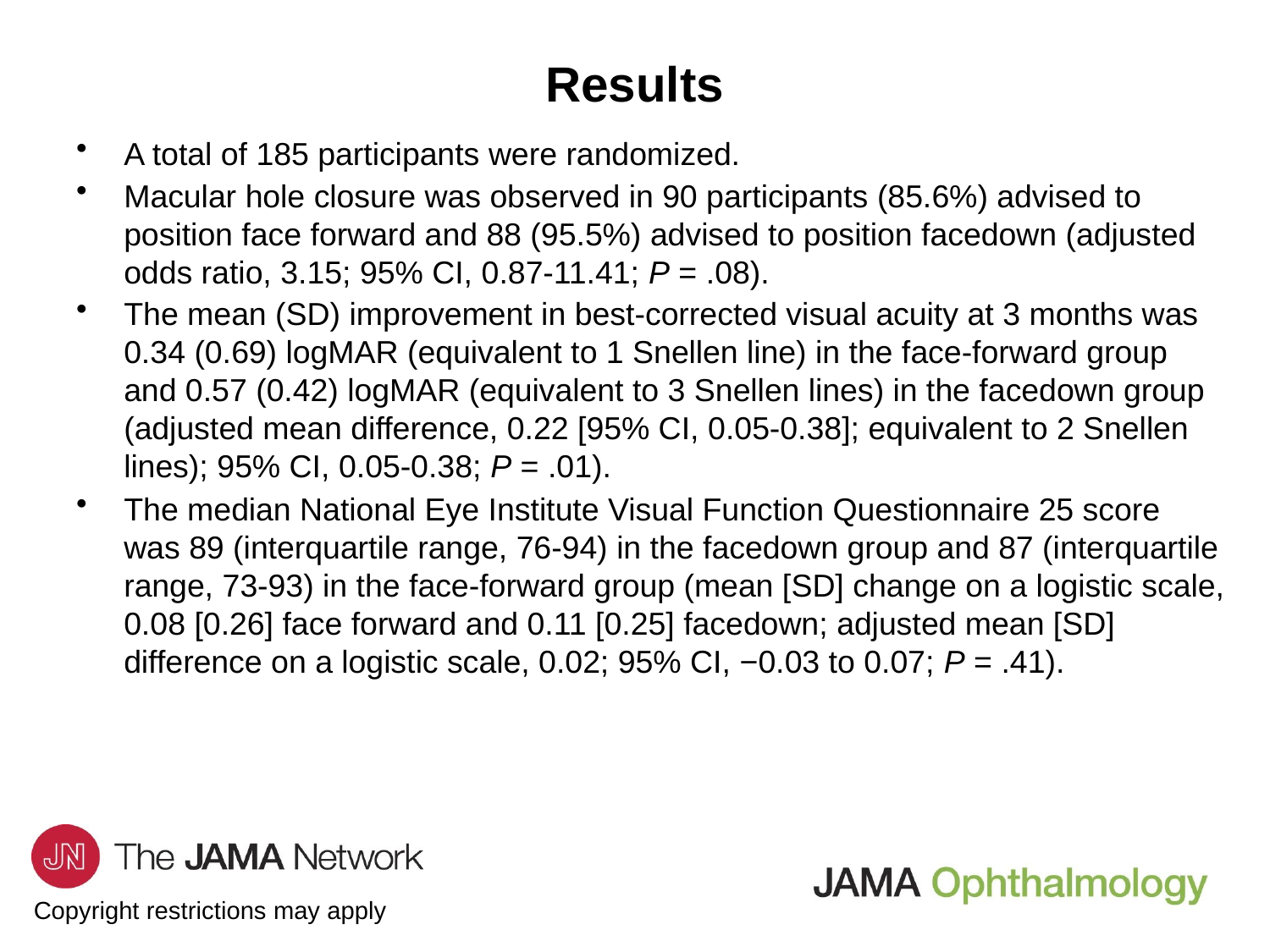

# Results
A total of 185 participants were randomized.
Macular hole closure was observed in 90 participants (85.6%) advised to position face forward and 88 (95.5%) advised to position facedown (adjusted odds ratio, 3.15; 95% CI, 0.87-11.41; P = .08).
The mean (SD) improvement in best-corrected visual acuity at 3 months was 0.34 (0.69) logMAR (equivalent to 1 Snellen line) in the face-forward group and 0.57 (0.42) logMAR (equivalent to 3 Snellen lines) in the facedown group (adjusted mean difference, 0.22 [95% CI, 0.05-0.38]; equivalent to 2 Snellen lines); 95% CI, 0.05-0.38; P = .01).
The median National Eye Institute Visual Function Questionnaire 25 score was 89 (interquartile range, 76-94) in the facedown group and 87 (interquartile range, 73-93) in the face-forward group (mean [SD] change on a logistic scale, 0.08 [0.26] face forward and 0.11 [0.25] facedown; adjusted mean [SD] difference on a logistic scale, 0.02; 95% CI, −0.03 to 0.07; P = .41).

## Slide 6
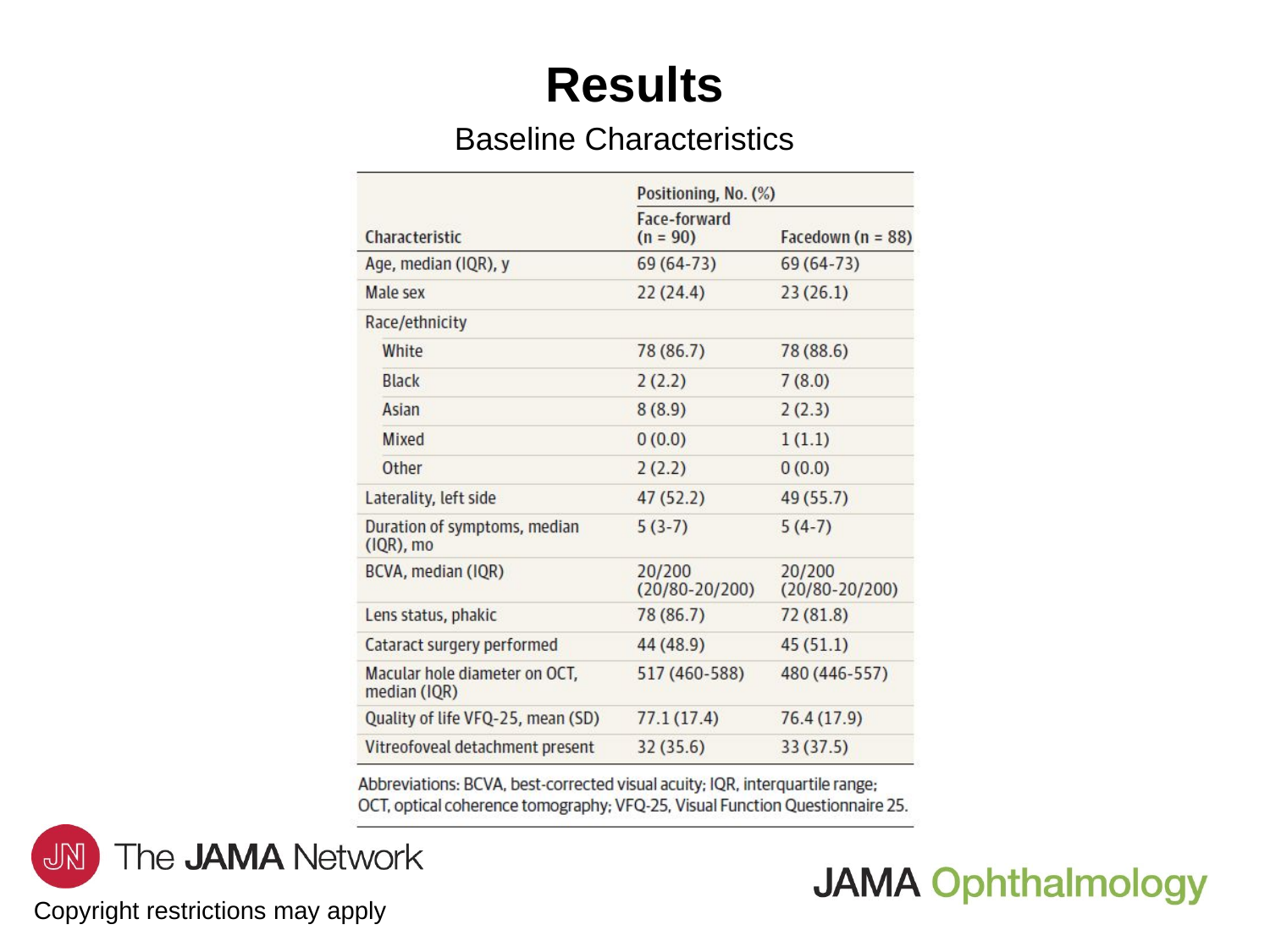

# Results
Baseline Characteristics

## Slide 7
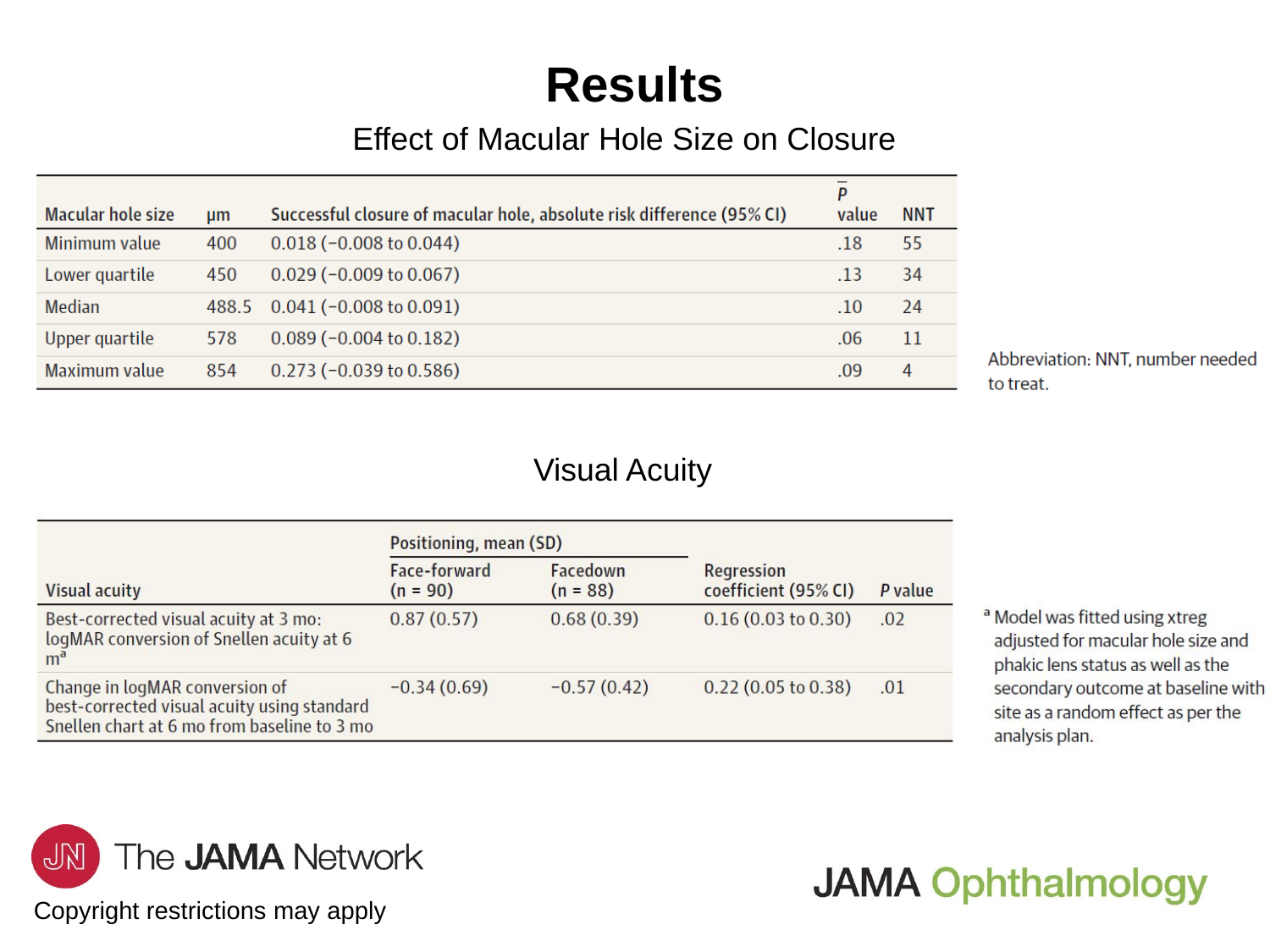

# Results
Effect of Macular Hole Size on Closure
Visual Acuity

## Slide 8
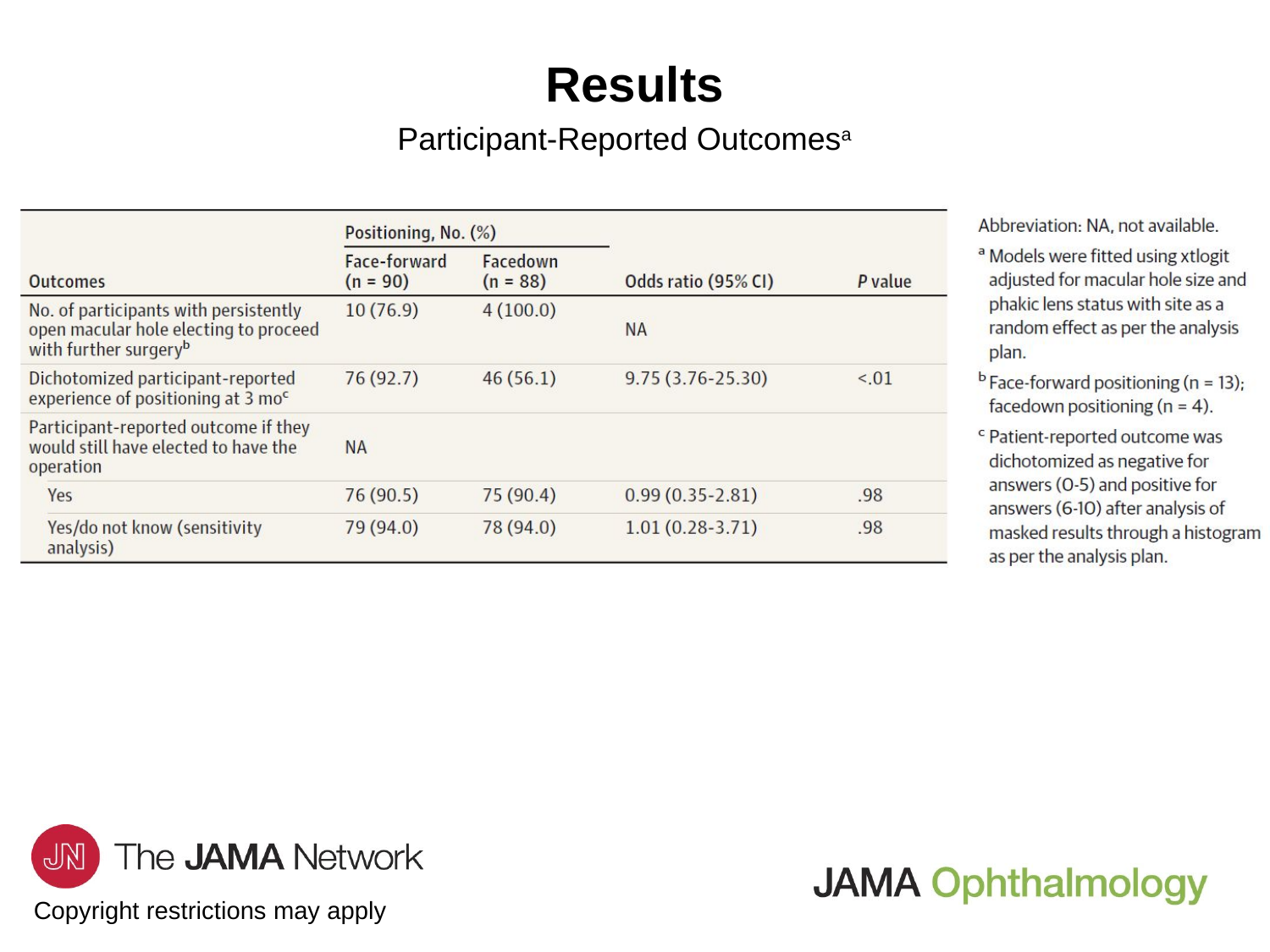

# Results
Participant-Reported Outcomesa

## Slide 9
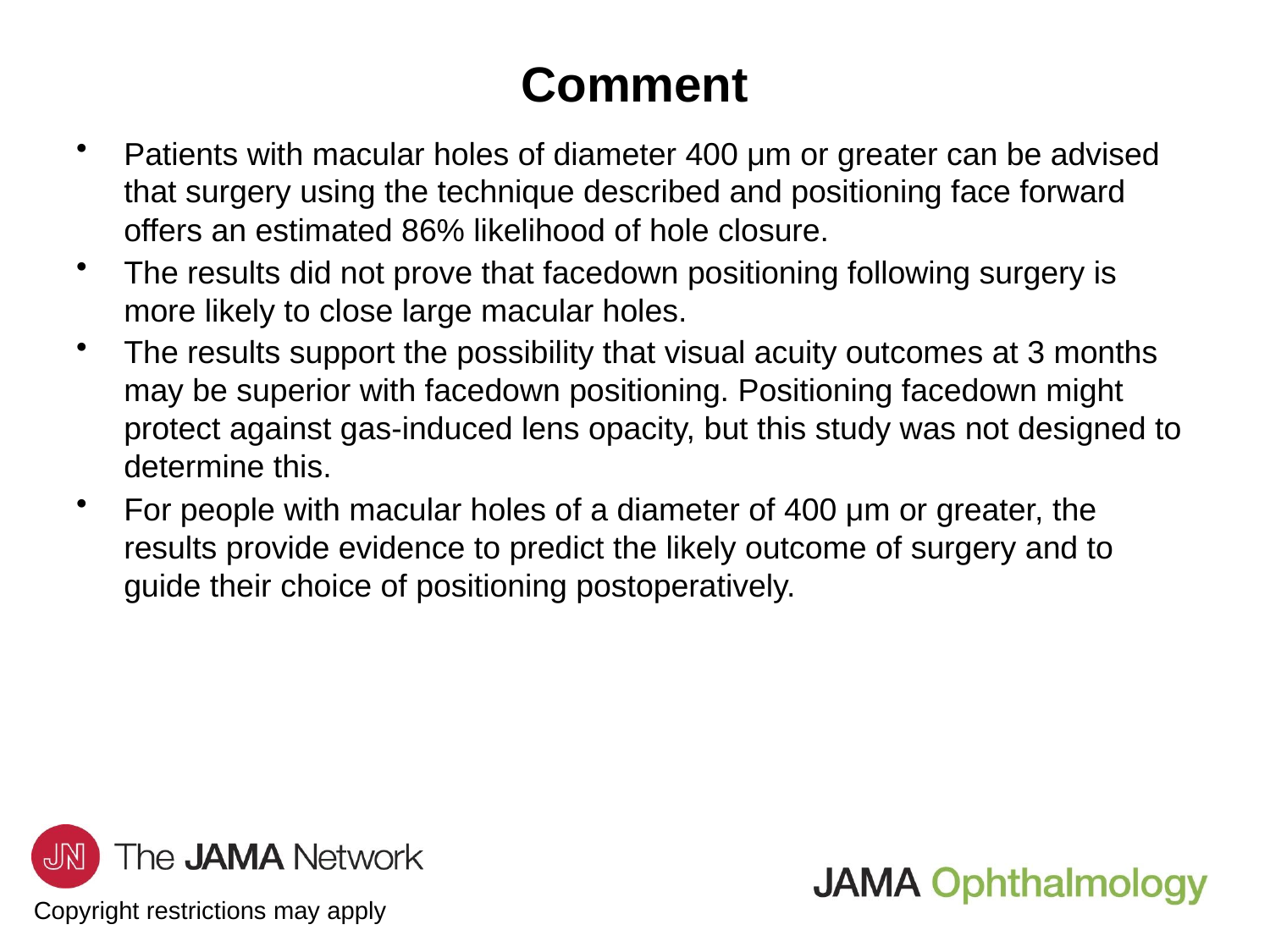

# Comment
Patients with macular holes of diameter 400 μm or greater can be advised that surgery using the technique described and positioning face forward offers an estimated 86% likelihood of hole closure.
The results did not prove that facedown positioning following surgery is more likely to close large macular holes.
The results support the possibility that visual acuity outcomes at 3 months may be superior with facedown positioning. Positioning facedown might protect against gas-induced lens opacity, but this study was not designed to determine this.
For people with macular holes of a diameter of 400 μm or greater, the results provide evidence to predict the likely outcome of surgery and to guide their choice of positioning postoperatively.

## Slide 10
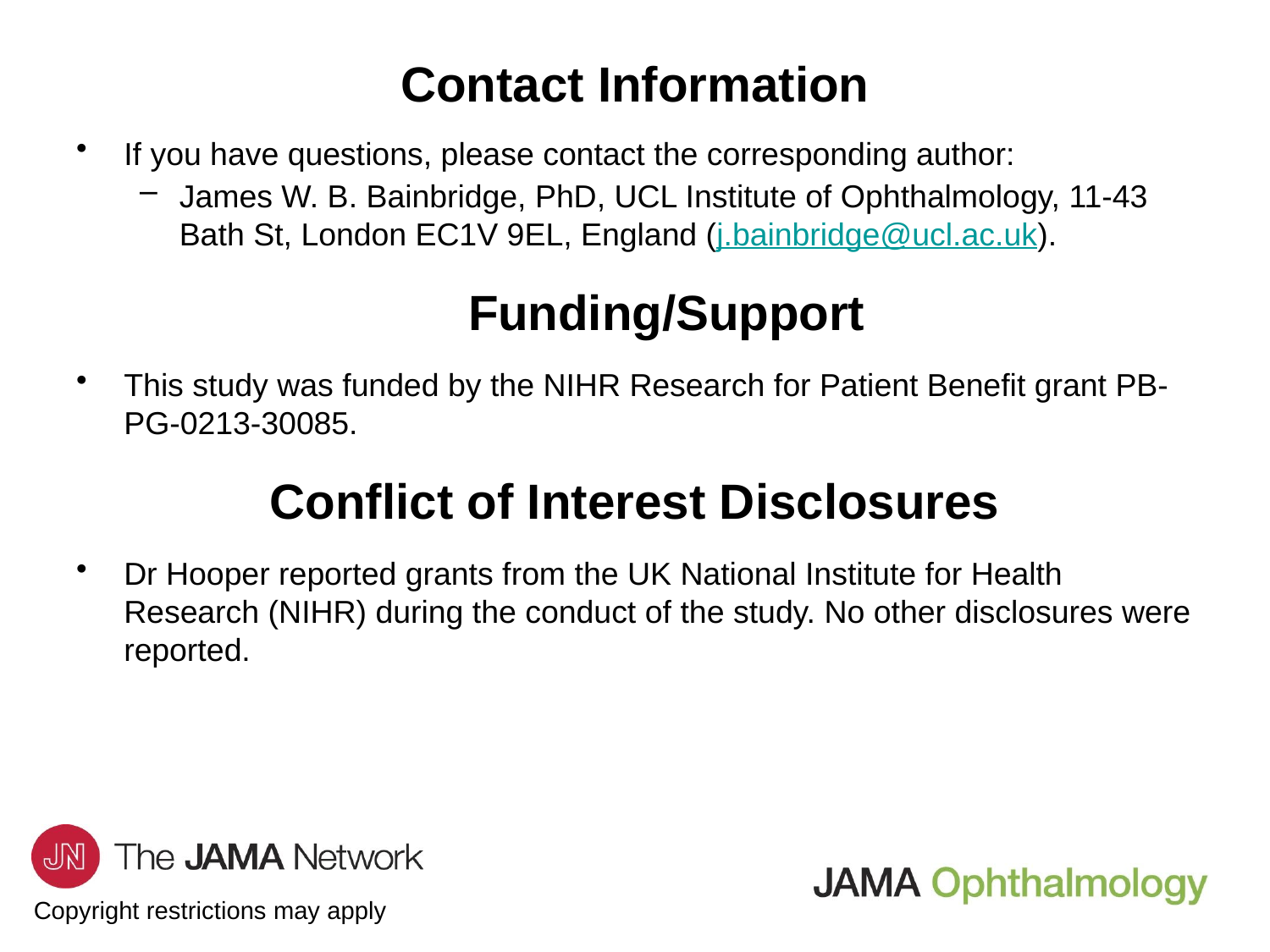

# Contact Information
If you have questions, please contact the corresponding author:
James W. B. Bainbridge, PhD, UCL Institute of Ophthalmology, 11-43 Bath St, London EC1V 9EL, England (j.bainbridge@ucl.ac.uk).
Funding/Support
This study was funded by the NIHR Research for Patient Benefit grant PB-PG-0213-30085.
Conflict of Interest Disclosures
Dr Hooper reported grants from the UK National Institute for Health Research (NIHR) during the conduct of the study. No other disclosures were reported.
